# Crystal structure of creatininium 5-(2,4-di­nitro­phen­yl)-1,3-di­methyl­barbiturate monohydrate: a potential anti­convulsant agent

**DOI:** 10.1107/S2056989016005247

**Published:** 2016-04-05

**Authors:** Ponnusamy Poornima Devi, Doraisamyraja Kalaivani

**Affiliations:** a PG and Research Department of Chemistry, Seethalakshmi Ramaswami College, Tiruchirappalli 620 002, Tamil Nadu, India

**Keywords:** crystal structure, creatinine, creatininium, 5-(2,4-di­nitro­phen­yl)-*N*,*N*-di­methyl­barbiturate, anti­convulsant activity, hydrogen bonding

## Abstract

In the anion of the title hydrated mol­ecular salt, C_4_H_8_N_3_O^+^·C_12_H_9_N_4_O_7_^−^·H_2_O [systematic name: 2-amino-1-methyl-4-oxo-4,5-di­hydro-1*H*-imidazol-3-ium 5-(2,4-di­nitro­phen­yl)-1,3-dimethyl-2,6-dioxo-1,2,3,6-tetra­hydro­pyrimidin-4-olate monohydrate], the 2,4-di­nitro­phenyl ring is inclined to the mean plane of the pyrimidine ring [r.m.s. deviation = 0.37 Å] by 43.24 (8)°. The five-membered ring of the creatininium cation (2-amino-1-methyl-4-oxo-4,5-di­hydro-1*H*-imidazol-3-ium) is essentially planar with an r.m.s. deviation of 0.015 Å. In the crystal, the anions and cations are linked *via* N—H⋯O hydrogen bonds, forming sheets parallel to the *ab* plane. The sheets are linked *via* O—H⋯O hydrogen bonds involving the water mol­ecule, forming a three-dimensional framework. Within the framework, there are C—H⋯O hydrogen bonds present. The title mol­ecular salt displays anti­convulsant and hypnotic activities.

## Abstract 2

The title mol­ecular salt exhibits anti­convulsant and hypnotic activities. In the crystal, the 5-(2,4-di­nitro­phen­yl)-*N*,*N*-di­methyl­barbiturate anion is linked to the creatininium cation by N—H⋯O hydrogen bonds, forming sheets parallel to the *ab* plane.

## Chemical context   

Creatinine is a breakdown product of creatine phosphate during metabolic activity in living systems (Ueda, 1964 ▸). Creatinine exists in both the amino and the imino tautomeric forms. Due to the presence of various groups, such as CH_3_, CH_2_, NH, NH_2_ and C=O, it can form C—H⋯O, N—H⋯O and O—H⋯O hydrogen bonds with other mol­ecules. Barbiturates are pyrimidine derivatives which exhibit their action by modulating the ion channels. Pyrimidine and its derivatives have been shown to be effective medications (Brown, 1962 ▸; Gauthier *et al.*, 1963 ▸; Shorvon, 2004 ▸; Jain *et al.*, 2006 ▸; Tripathi, 2009 ▸). In this context, a number of pharmacologically active mol­ecular salts with different barbiturate entities and cationic counter parts have been described (see for example: Rajamani & Kalaivani, 2015 ▸; Gomathi & Kalaivani, 2015 ▸). Herein, we describe the synthesis and crystal structure of the title mol­ecular salt, which has been shown to exhibit anti­convulsant and hypnotic activities.
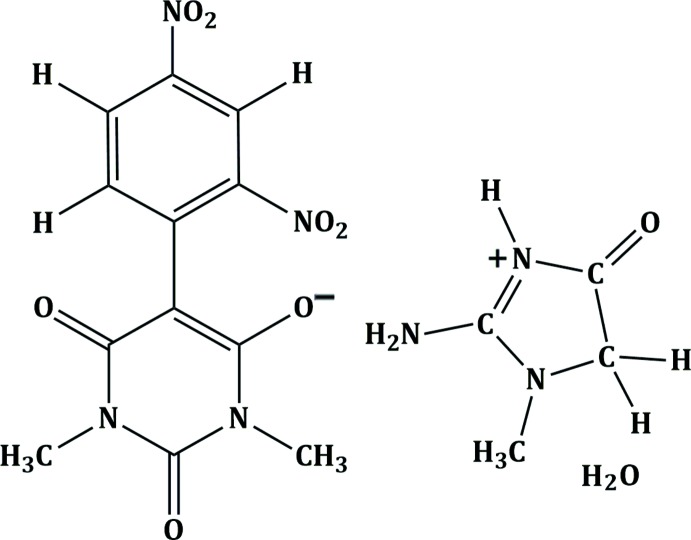


## Structural commentary   

**Figure 1:**
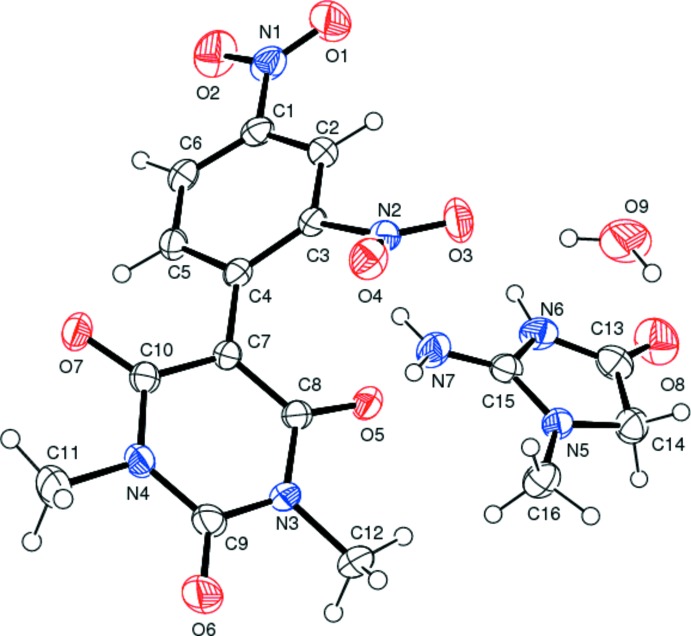
The mol­ecular structure of the title mol­ecular salt, with atom labelling. Displacement ellipsoids are drawn at the 40% probability level.

The structure of the title mol­ecular salt is illustrated in Fig. 1 ▸. The bond lengths and bond angles are normal and comparable with those observed in related barbiturates (Sridevi & Kalaivani, 2012 ▸; Gunaseelan & Doraisamyraja, 2014 ▸). The five-membered ring of the creatininium (2-amino-1-methyl-4-oxo-4,5-di­hydro-1*H*-imidazol-3-ium) cation is essentially planar with an r.m.s. deviation of 0.015 Å. In the anion, the 2,4-di­nitro­phenyl ring is inclined to the mean plane of the pyrimidine ring (r.m.s. deviation = 0.37 Å) by 43.24 (8)°. The nitro group *ortho* with respect to ring junction is inclined to the benzene ring to which it is attached by 37.6 (2)°, while the nitro group *para* with respect to the ring junction is inclined to the benzene ring by 7.4 (3)°. The different dihedral angles imply that though two nitro groups are involved in delocalizing the negative charge on the oxygen atom of barbiturate ion, the *para* nitro group is more effective than the *ortho* nitro group.

## Supra­molecular features   

**Figure 2:**
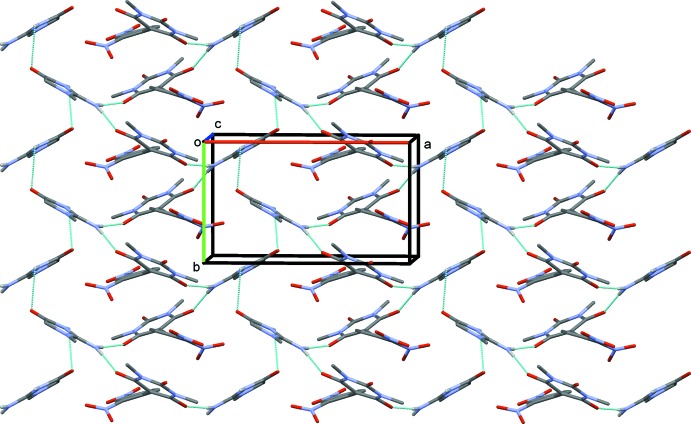
A view along the *c* axis of the crystal packing of the title mol­ecular salt. The hydrogen bonds are shown as dashed lines (see Table 1 ▸), and the water mol­ecule and C-bound H atoms have been omitted for clarity.

**Figure 3:**
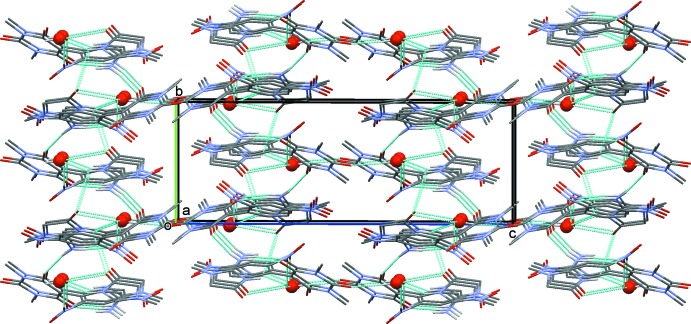
A view along the *a* axis of the crystal packing of the title mol­ecular salt. The hydrogen bonds are shown as dashed lines (Table 1 ▸). The C-bound H atoms have been omitted for clarity, and the water mol­ecules are shown as red balls.

In the crystal, the anion and cation are linked *via* N—H⋯O hydrogen bonds, forming sheets parallel to the *ab* plane (Fig. 2 ▸ and Table 1 ▸). The sheets are linked *via* O—H⋯O hydrogen bonds involving the water mol­ecule, forming a three-dimensional framework (Fig. 3 ▸ and Table 1 ▸). Within the framework, there are C—H⋯O hydrogen bonds present (Table 1 ▸).

## Database survey   

A search of the Cambridge Structural Database (CSD, Version 53.7, last update February 2016; Groom & Allen, 2014 ▸) for the title anion as sub-structure gave 17 hits, of which five involve 5-(2,4-di­nitro­phen­yl)-1,3-di­methyl­barbiturate and organic cations. They include the mol­ecular salts of 3-amino­pyridinium (CSD refcode QUNRAU; Kalaivani & Sridevi, 2015*a* ▸), 4-amino­pyridinium (QUNROI; Kalaivani & Sridevi, 2015*b* ▸), *N*,*N*-di­ethyl­ethano­lammonium (QUNRUO; Kalaivani & Sridevi, 2015*c* ▸), tri­methyl­ammonium (CORWUD; Gunaseelan & Doraisamyraja, 2014 ▸) and 2-methyl­pyridinium (YAVSOF; Sridevi & Kalaivani, 2012 ▸). In the anions, the benzene ring is inclined to the mean plane of the pyrimidine ring by dihedral angles varying from *ca* 39.0 to 50.5°. The *ortho* nitro group is inclined to the benzene ring by dihedral angles varying from *ca* 2.4 to 5.8°, and the *para* nitro group is inclined to the benzene ring by a much larger angle, varying between *ca* 37.2 and 42.6°. Similar observations were made for the conformation of the barbiturate anion in the title mol­ecular salt.

## Biological activity   

Epilepsy (convulsion) is one of the most common neurodegenerative disorder affecting at least 50 million people worldwide. Brain dysfunction due to different causes leads to epilepsy (Fisher *et al.*, 2005 ▸). Barbiturates have a pyrimidone ring system. From their introduction into clinical practice at the beginning of the 20th century until recent years, they have occupied a vital place in the pharmacopoeia as CNS drugs (Yadav, 2004 ▸). The anti­convulsant activity of the synthesized barbiturate has been measured by employing the Maximal Electro Shock method (Kulkarni, 1999 ▸). In the present investigation, the title mol­ecular salt reduces the clonus phase of convulsion to a greater extent than other phases of convulsion (flexion, extension and stupor) even at low dosage (25 mg kg^−1^) and hence may be used in the future for controlling myoclonic epilepsy of infants. The therapeutic dose induces hypnosis in albino mice. Acute toxicity tests have also been carried out according to OECD guidelines on albino mice (LD_50_ >1000 mg kg ^−1^; falls under class 4). The animals did not show any indication of behavioural changes after testing with the title mol­ecular salt. The high safety margin reveals its significance as a potential anti­convulsant agent.

## Synthesis and crystallization   

Di­nitro­chloro­benzene (2.02 g, 0.01 mol) was dissolved in 20 ml of absolute alcohol. To this 1.56 g (0.01mol) of 1,3-di­methyl­barbituric acid was added and the temperature of the mixture was raised to 323 K. To this mixture 1.13 g (0.01 mol) of creatinine in 20 ml of absolute alcohol was added. This mixture was shaken well for 2–5 h and kept as such at 298 K for 2 d. On standing, a maroon-red-coloured solid came out from the solution. The solid was ground to a fine powder, washed with absolute alcohol and dried with ether and then recrystallized from absolute alcohol. The solution was left to stand and maroon-red block-shaped crystals were obtained after two weeks. The crystals were harvested and air dried (yield: 80%; m.p. 483 K).

## Refinement   

Crystal data, data collection and structure refinement details are summarized in Table 2 ▸. The NH H atoms were located from a difference Fourier map and freely refined. The water mol­ecule H atoms were also located from a difference Fourier map and refined with *U*_iso_(H) = 1.5*U*_eq_(O). The C-bound H atoms were included in calculated positions and treated as riding atoms: C—H = 0.93–0.97 Å with *U*_iso_(H) = 1.5*U*_eq_(C-meth­yl) and 1.2*U*_eq_(C) for other H atoms.

## Supplementary Material

Crystal structure: contains datablock(s) global, I. DOI: 10.1107/S2056989016005247/su5291sup1.cif

Structure factors: contains datablock(s) I. DOI: 10.1107/S2056989016005247/su5291Isup2.hkl

CCDC reference: 1060400

Additional supporting information:  crystallographic information; 3D view; checkCIF report

## Figures and Tables

**Table 1:** Hydrogen-bond geometry (Å, °)

*D*—H⋯*A*	*D*—H	H⋯*A*	*D*⋯*A*	*D*—H⋯*A*
N7—H7*N*1⋯O5	0.85 (3)	1.96 (3)	2.800 (2)	171 (2)
N7—H7*N*2⋯O7^i^	0.82 (3)	1.95 (3)	2.749 (2)	165 (3)
N6—H6*N*⋯O1*W* ^ii^	0.84 (3)	2.05 (3)	2.767 (2)	142 (2)
O1*W*—H1*WA*⋯O6^iii^	0.81 (4)	1.99 (4)	2.792 (2)	166 (4)
O1*W*—H1*WB*⋯O3	0.79 (4)	2.47 (4)	3.083 (3)	136 (4)
O1*W*—H1*WB*⋯O8^iv^	0.79 (4)	2.61 (4)	3.080 (3)	120 (4)
C12—H12*C*⋯O5^iii^	0.96	2.57	3.483 (3)	159
C14—H14*B*⋯O1^ii^	0.97	2.44	3.270 (3)	144
C16—H16*C*⋯O5	0.96	2.54	3.248 (2)	131

Symmetry codes: (i) 
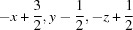
; (ii) 
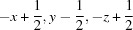
; (iii) 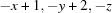; (iv) 
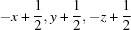
.

**Table 2:** Experimental details

Crystal data	
Chemical formula	C_4_H_8_N_3_O^+^·C_12_H_9_N_4_O_7_^−^·H_2_O
*M*_r_	453.38
Crystal system, space group	Monoclinic, *P*2_1_/*n*
Temperature (K)	293
*a*, *b*, *c* (Å)	12.6926 (3), 7.3093 (2), 20.6213 (5)
β (°)	100.420 (4)
*V* (Å^3^)	1881.57 (9)
*Z*	4
Radiation type	Mo *K*α
μ (mm^−1^)	0.13
Crystal size (mm)	0.35 × 0.30 × 0.25

Data collection	
Diffractometer	Bruker Kappa APEXII CCD Diffractometer
Absorption correction	Multi-scan (*SADABS*; Bruker, 2004 ▸)
*T*_min_, *T*_max_	0.954, 0.969
No. of measured, independent and observed [*I* > 2σ(*I*)] reflections	32561, 5338, 3586
*R*_int_	0.037
(sin θ/λ)_max_ (Å^−1^)	0.699

Refinement	
*R*[*F* ^2^ > 2σ(*F* ^2^)], *wR*(*F* ^2^), *S*	0.051, 0.137, 1.03
No. of reflections	5338
No. of parameters	310
H-atom treatment	H atoms treated by a mixture of independent and constrained refinement
Δρ_max_, Δρ_min_ (e Å^−3^)	0.40, −0.36

Computer programs: *APEX2* *SAINT* and *XPREP* (Bruker, 2004 ▸), *SIR92* (Altomare *et al.*, 1993 ▸), *ORTEP-3 for Windows* (Farrugia, 2012 ▸), *Mercury* (Macrae *et al.*, 2008 ▸), *SHELXL2014/7* (Sheldrick, 2015 ▸) and *PLATON* (Spek, 2009 ▸).

## supplementary crystallographic information

### Crystal data

**Table:**

C_4_H_8_N_3_O^+^·C_12_H_9_N_4_O_7_^−^·H_2_O	*F*(000) = 944
*M**_r_* = 453.38	*D*_x_ = 1.600 Mg m^−^^3^
Monoclinic, *P*2_1_/*n*	Mo *K*α radiation, λ = 0.71073 Å
*a* = 12.6926 (3) Å	Cell parameters from 7465 reflections
*b* = 7.3093 (2) Å	θ = 3.0–26.5°
*c* = 20.6213 (5) Å	µ = 0.13 mm^−^^1^
β = 100.420 (4)°	*T* = 293 K
*V* = 1881.57 (9) Å^3^	Block, brown
*Z* = 4	0.35 × 0.30 × 0.25 mm

### Data collection

**Table:**

Bruker Kappa APEXII CCD Diffractometer	5338 independent reflections
Radiation source: fine-focus sealed tube	3586 reflections with *I* > 2σ(*I*)
Graphite monochromator	*R*_int_ = 0.037
ω and φ scan	θ_max_ = 29.8°, θ_min_ = 3.0°
Absorption correction: multi-scan (*SADABS*; Bruker, 2004)	*h* = −17→17
*T*_min_ = 0.954, *T*_max_ = 0.969	*k* = −9→10
32561 measured reflections	*l* = −28→28

### Refinement

**Table:**

Refinement on *F*^2^	Primary atom site location: structure-invariant direct methods
Least-squares matrix: full	Secondary atom site location: difference Fourier map
*R*[*F*^2^ > 2σ(*F*^2^)] = 0.051	Hydrogen site location: mixed
*wR*(*F*^2^) = 0.137	H atoms treated by a mixture of independent and constrained refinement
*S* = 1.03	*w* = 1/[σ^2^(*F*_o_^2^) + (0.0513*P*)^2^ + 1.1255*P*] where *P* = (*F*_o_^2^ + 2*F*_c_^2^)/3
5338 reflections	(Δ/σ)_max_ < 0.001
310 parameters	Δρ_max_ = 0.40 e Å^−^^3^
0 restraints	Δρ_min_ = −0.36 e Å^−^^3^

### Special details

**Table:**

Geometry. All esds (except the esd in the dihedral angle between two l.s. planes) are estimated using the full covariance matrix. The cell esds are taken into account individually in the estimation of esds in distances, angles and torsion angles; correlations between esds in cell parameters are only used when they are defined by crystal symmetry. An approximate (isotropic) treatment of cell esds is used for estimating esds involving l.s. planes.

### Fractional atomic coordinates and isotropic or equivalent isotropic displacement parameters (Å^2^)

**Table:**

	*x*	*y*	*z*	*U*_iso_*/*U*_eq_	
O1	0.51360 (14)	1.1815 (3)	0.40647 (8)	0.0644 (5)	
O2	0.66430 (15)	1.0674 (3)	0.45047 (7)	0.0649 (5)	
O3	0.42214 (11)	1.1907 (2)	0.16944 (8)	0.0524 (4)	
O4	0.54626 (11)	1.31323 (19)	0.12732 (7)	0.0418 (3)	
O5	0.57211 (10)	0.91799 (18)	0.10996 (6)	0.0350 (3)	
O6	0.81530 (12)	0.9893 (3)	−0.02112 (7)	0.0586 (5)	
O7	0.90227 (10)	1.1964 (2)	0.18675 (6)	0.0403 (3)	
O8	0.15322 (15)	0.4142 (3)	0.20438 (9)	0.0682 (5)	
N1	0.60178 (14)	1.1239 (3)	0.40331 (8)	0.0429 (4)	
N2	0.51596 (12)	1.2223 (2)	0.16920 (7)	0.0326 (3)	
N3	0.69565 (12)	0.9495 (2)	0.04581 (7)	0.0321 (3)	
N4	0.85766 (12)	1.0943 (2)	0.08271 (7)	0.0345 (4)	
N5	0.32746 (12)	0.6035 (2)	0.11207 (7)	0.0337 (3)	
N6	0.31529 (15)	0.5560 (3)	0.21465 (9)	0.0421 (4)	
H6N	0.3318 (19)	0.566 (3)	0.2560 (13)	0.054 (7)*	
N7	0.46476 (15)	0.7122 (3)	0.19231 (9)	0.0423 (4)	
H7N1	0.4961 (19)	0.765 (3)	0.1642 (13)	0.053 (7)*	
H7N2	0.495 (2)	0.715 (4)	0.2309 (14)	0.062 (8)*	
C1	0.63499 (15)	1.1227 (3)	0.33968 (8)	0.0314 (4)	
C2	0.56212 (14)	1.1645 (3)	0.28472 (9)	0.0312 (4)	
H2	0.4924	1.1977	0.2876	0.037*	
C3	0.59481 (13)	1.1560 (2)	0.22486 (8)	0.0267 (3)	
C4	0.69704 (13)	1.1026 (2)	0.21720 (8)	0.0248 (3)	
C5	0.76756 (14)	1.0670 (2)	0.27580 (8)	0.0292 (4)	
H5	0.8379	1.0361	0.2737	0.035*	
C6	0.73800 (15)	1.0754 (3)	0.33611 (9)	0.0319 (4)	
H6	0.7871	1.0494	0.3742	0.038*	
C7	0.72996 (13)	1.0733 (2)	0.15415 (8)	0.0254 (3)	
C8	0.66101 (13)	0.9796 (2)	0.10494 (8)	0.0265 (3)	
C9	0.79069 (15)	1.0101 (3)	0.03282 (9)	0.0364 (4)	
C10	0.83323 (13)	1.1237 (2)	0.14538 (8)	0.0289 (4)	
C11	0.96085 (17)	1.1545 (4)	0.06976 (11)	0.0524 (6)	
H11A	0.9979	1.2210	0.1072	0.079*	
H11B	0.9503	1.2323	0.0316	0.079*	
H11C	1.0026	1.0499	0.0619	0.079*	
C12	0.62720 (17)	0.8444 (3)	−0.00485 (10)	0.0446 (5)	
H12A	0.6045	0.7346	0.0142	0.067*	
H12B	0.6662	0.8131	−0.0390	0.067*	
H12C	0.5656	0.9161	−0.0232	0.067*	
C13	0.22262 (18)	0.4829 (3)	0.18063 (11)	0.0444 (5)	
C14	0.22732 (16)	0.5085 (3)	0.10967 (10)	0.0429 (5)	
H14A	0.2274	0.3919	0.0872	0.051*	
H14B	0.1675	0.5814	0.0877	0.051*	
C15	0.37450 (15)	0.6299 (3)	0.17320 (9)	0.0334 (4)	
C16	0.37034 (17)	0.6532 (3)	0.05467 (9)	0.0404 (5)	
H16A	0.3160	0.6388	0.0161	0.061*	
H16B	0.4301	0.5757	0.0513	0.061*	
H16C	0.3935	0.7784	0.0583	0.061*	
O1W	0.21039 (19)	0.9763 (4)	0.15794 (9)	0.0809 (7)	
H1WA	0.192 (3)	0.992 (6)	0.118 (2)	0.121*	
H1WB	0.273 (3)	0.976 (6)	0.171 (2)	0.121*	

### Atomic displacement parameters (Å^2^)

**Table:**

	*U*^11^	*U*^22^	*U*^33^	*U*^12^	*U*^13^	*U*^23^
O1	0.0526 (10)	0.1010 (14)	0.0450 (9)	0.0003 (10)	0.0227 (8)	−0.0113 (9)
O2	0.0679 (11)	0.1012 (15)	0.0246 (7)	−0.0079 (10)	0.0059 (7)	0.0078 (8)
O3	0.0254 (7)	0.0828 (12)	0.0468 (9)	0.0056 (7)	0.0006 (6)	−0.0017 (8)
O4	0.0438 (8)	0.0430 (8)	0.0357 (7)	0.0047 (6)	−0.0008 (6)	0.0076 (6)
O5	0.0309 (7)	0.0410 (7)	0.0322 (7)	−0.0071 (5)	0.0032 (5)	−0.0064 (6)
O6	0.0440 (8)	0.1058 (14)	0.0280 (7)	0.0054 (9)	0.0118 (6)	−0.0102 (8)
O7	0.0288 (6)	0.0586 (9)	0.0318 (7)	−0.0090 (6)	0.0007 (5)	−0.0060 (6)
O8	0.0617 (11)	0.0856 (13)	0.0648 (11)	−0.0101 (10)	0.0316 (9)	0.0136 (10)
N1	0.0469 (10)	0.0557 (11)	0.0278 (8)	−0.0132 (8)	0.0108 (7)	−0.0056 (8)
N2	0.0297 (8)	0.0379 (9)	0.0285 (8)	0.0062 (6)	0.0003 (6)	−0.0053 (6)
N3	0.0295 (7)	0.0421 (9)	0.0231 (7)	0.0029 (6)	0.0009 (6)	−0.0081 (6)
N4	0.0249 (7)	0.0524 (10)	0.0271 (7)	0.0021 (7)	0.0068 (6)	−0.0017 (7)
N5	0.0316 (8)	0.0446 (9)	0.0253 (7)	−0.0003 (7)	0.0059 (6)	−0.0001 (6)
N6	0.0536 (11)	0.0490 (10)	0.0251 (8)	0.0033 (8)	0.0109 (7)	0.0023 (7)
N7	0.0430 (10)	0.0549 (11)	0.0266 (9)	−0.0013 (8)	0.0001 (7)	−0.0003 (8)
C1	0.0373 (9)	0.0351 (10)	0.0222 (8)	−0.0065 (8)	0.0067 (7)	−0.0042 (7)
C2	0.0280 (8)	0.0358 (9)	0.0304 (9)	−0.0004 (7)	0.0070 (7)	−0.0045 (7)
C3	0.0252 (8)	0.0297 (9)	0.0237 (8)	0.0006 (7)	0.0000 (6)	−0.0031 (6)
C4	0.0258 (8)	0.0236 (8)	0.0238 (8)	−0.0015 (6)	0.0014 (6)	−0.0021 (6)
C5	0.0261 (8)	0.0329 (9)	0.0275 (8)	0.0007 (7)	0.0019 (6)	0.0007 (7)
C6	0.0330 (9)	0.0361 (10)	0.0238 (8)	−0.0028 (7)	−0.0022 (7)	0.0006 (7)
C7	0.0243 (8)	0.0291 (8)	0.0220 (8)	0.0017 (6)	0.0017 (6)	−0.0017 (6)
C8	0.0267 (8)	0.0283 (9)	0.0236 (8)	0.0035 (7)	0.0022 (6)	−0.0012 (6)
C9	0.0314 (9)	0.0527 (12)	0.0248 (8)	0.0095 (8)	0.0042 (7)	−0.0017 (8)
C10	0.0264 (8)	0.0337 (9)	0.0256 (8)	0.0042 (7)	0.0021 (6)	0.0003 (7)
C11	0.0337 (10)	0.0796 (17)	0.0469 (12)	−0.0037 (11)	0.0153 (9)	−0.0028 (12)
C12	0.0435 (11)	0.0550 (13)	0.0320 (10)	0.0030 (10)	−0.0024 (8)	−0.0169 (9)
C13	0.0464 (12)	0.0476 (12)	0.0426 (11)	0.0034 (10)	0.0170 (9)	0.0039 (9)
C14	0.0368 (10)	0.0555 (13)	0.0372 (10)	−0.0052 (9)	0.0090 (8)	−0.0014 (9)
C15	0.0386 (10)	0.0349 (10)	0.0266 (9)	0.0081 (8)	0.0053 (7)	−0.0001 (7)
C16	0.0459 (11)	0.0476 (12)	0.0271 (9)	−0.0070 (9)	0.0051 (8)	0.0031 (8)
O1W	0.0862 (15)	0.1218 (18)	0.0345 (9)	0.0251 (15)	0.0102 (10)	−0.0039 (11)

### Geometric parameters (Å, º)

**Table:**

O1—N1	1.208 (2)	C1—C6	1.367 (3)
O2—N1	1.211 (2)	C2—C3	1.373 (2)
O3—N2	1.214 (2)	C2—H2	0.9300
O4—N2	1.207 (2)	C3—C4	1.391 (2)
O5—C8	1.237 (2)	C4—C5	1.392 (2)
O6—C9	1.218 (2)	C4—C7	1.452 (2)
O7—C10	1.228 (2)	C5—C6	1.364 (2)
O8—C13	1.193 (3)	C5—H5	0.9300
N1—C1	1.449 (2)	C6—H6	0.9300
N2—C3	1.462 (2)	C7—C8	1.394 (2)
N3—C9	1.356 (2)	C7—C10	1.404 (2)
N3—C8	1.387 (2)	C11—H11A	0.9600
N3—C12	1.452 (2)	C11—H11B	0.9600
N4—C9	1.358 (2)	C11—H11C	0.9600
N4—C10	1.399 (2)	C12—H12A	0.9600
N4—C11	1.452 (2)	C12—H12B	0.9600
N5—C15	1.308 (2)	C12—H12C	0.9600
N5—C16	1.436 (2)	C13—C14	1.487 (3)
N5—C14	1.441 (2)	C14—H14A	0.9700
N6—C15	1.349 (3)	C14—H14B	0.9700
N6—C13	1.365 (3)	C16—H16A	0.9600
N6—H6N	0.84 (3)	C16—H16B	0.9600
N7—C15	1.291 (3)	C16—H16C	0.9600
N7—H7N1	0.85 (3)	O1W—H1WA	0.81 (4)
N7—H7N2	0.82 (3)	O1W—H1WB	0.79 (4)
C1—C2	1.362 (2)		
			
O1—N1—O2	123.65 (18)	C10—C7—C4	120.21 (15)
O1—N1—C1	118.37 (17)	O5—C8—N3	117.07 (15)
O2—N1—C1	117.98 (18)	O5—C8—C7	125.47 (15)
O4—N2—O3	123.20 (16)	N3—C8—C7	117.45 (15)
O4—N2—C3	118.87 (15)	O6—C9—N3	121.57 (18)
O3—N2—C3	117.82 (16)	O6—C9—N4	121.32 (18)
C9—N3—C8	123.82 (15)	N3—C9—N4	117.11 (15)
C9—N3—C12	117.94 (15)	O7—C10—N4	117.31 (16)
C8—N3—C12	118.23 (15)	O7—C10—C7	126.12 (16)
C9—N4—C10	123.82 (15)	N4—C10—C7	116.53 (15)
C9—N4—C11	117.39 (16)	N4—C11—H11A	109.5
C10—N4—C11	118.79 (16)	N4—C11—H11B	109.5
C15—N5—C16	125.59 (17)	H11A—C11—H11B	109.5
C15—N5—C14	110.50 (15)	N4—C11—H11C	109.5
C16—N5—C14	123.85 (16)	H11A—C11—H11C	109.5
C15—N6—C13	111.00 (17)	H11B—C11—H11C	109.5
C15—N6—H6N	122.8 (17)	N3—C12—H12A	109.5
C13—N6—H6N	125.9 (17)	N3—C12—H12B	109.5
C15—N7—H7N1	120.1 (17)	H12A—C12—H12B	109.5
C15—N7—H7N2	122.5 (19)	N3—C12—H12C	109.5
H7N1—N7—H7N2	117 (2)	H12A—C12—H12C	109.5
C2—C1—C6	121.62 (16)	H12B—C12—H12C	109.5
C2—C1—N1	119.16 (17)	O8—C13—N6	125.8 (2)
C6—C1—N1	119.21 (16)	O8—C13—C14	128.4 (2)
C1—C2—C3	117.90 (16)	N6—C13—C14	105.78 (17)
C1—C2—H2	121.0	N5—C14—C13	102.66 (16)
C3—C2—H2	121.0	N5—C14—H14A	111.2
C2—C3—C4	123.67 (16)	C13—C14—H14A	111.2
C2—C3—N2	114.55 (15)	N5—C14—H14B	111.2
C4—C3—N2	121.60 (15)	C13—C14—H14B	111.2
C3—C4—C5	114.87 (15)	H14A—C14—H14B	109.1
C3—C4—C7	124.65 (15)	N7—C15—N5	126.03 (18)
C5—C4—C7	120.38 (15)	N7—C15—N6	123.96 (18)
C6—C5—C4	122.91 (16)	N5—C15—N6	110.00 (18)
C6—C5—H5	118.5	N5—C16—H16A	109.5
C4—C5—H5	118.5	N5—C16—H16B	109.5
C5—C6—C1	118.95 (16)	H16A—C16—H16B	109.5
C5—C6—H6	120.5	N5—C16—H16C	109.5
C1—C6—H6	120.5	H16A—C16—H16C	109.5
C8—C7—C10	120.84 (15)	H16B—C16—H16C	109.5
C8—C7—C4	118.68 (15)	H1WA—O1W—H1WB	115 (4)
			
O1—N1—C1—C2	−8.0 (3)	C10—C7—C8—N3	−4.2 (2)
O2—N1—C1—C2	172.15 (19)	C4—C7—C8—N3	−178.39 (15)
O1—N1—C1—C6	173.55 (19)	C8—N3—C9—O6	−175.85 (19)
O2—N1—C1—C6	−6.3 (3)	C12—N3—C9—O6	5.2 (3)
C6—C1—C2—C3	0.7 (3)	C8—N3—C9—N4	4.5 (3)
N1—C1—C2—C3	−177.76 (16)	C12—N3—C9—N4	−174.44 (17)
C1—C2—C3—C4	1.8 (3)	C10—N4—C9—O6	179.29 (19)
C1—C2—C3—N2	−173.48 (16)	C11—N4—C9—O6	−1.5 (3)
O4—N2—C3—C2	139.47 (17)	C10—N4—C9—N3	−1.0 (3)
O3—N2—C3—C2	−36.7 (2)	C11—N4—C9—N3	178.17 (19)
O4—N2—C3—C4	−35.9 (2)	C9—N4—C10—O7	177.77 (18)
O3—N2—C3—C4	147.96 (17)	C11—N4—C10—O7	−1.4 (3)
C2—C3—C4—C5	−3.4 (3)	C9—N4—C10—C7	−4.6 (3)
N2—C3—C4—C5	171.52 (15)	C11—N4—C10—C7	176.16 (18)
C2—C3—C4—C7	172.93 (17)	C8—C7—C10—O7	−175.41 (17)
N2—C3—C4—C7	−12.2 (3)	C4—C7—C10—O7	−1.3 (3)
C3—C4—C5—C6	2.8 (3)	C8—C7—C10—N4	7.2 (2)
C7—C4—C5—C6	−173.66 (17)	C4—C7—C10—N4	−178.70 (15)
C4—C5—C6—C1	−0.7 (3)	C15—N6—C13—O8	−177.2 (2)
C2—C1—C6—C5	−1.2 (3)	C15—N6—C13—C14	2.4 (2)
N1—C1—C6—C5	177.25 (17)	C15—N5—C14—C13	0.1 (2)
C3—C4—C7—C8	−43.0 (2)	C16—N5—C14—C13	177.41 (18)
C5—C4—C7—C8	133.16 (17)	O8—C13—C14—N5	178.1 (2)
C3—C4—C7—C10	142.84 (18)	N6—C13—C14—N5	−1.5 (2)
C5—C4—C7—C10	−41.0 (2)	C16—N5—C15—N7	3.3 (3)
C9—N3—C8—O5	179.40 (17)	C14—N5—C15—N7	−179.4 (2)
C12—N3—C8—O5	−1.7 (2)	C16—N5—C15—N6	−175.89 (18)
C9—N3—C8—C7	−1.9 (3)	C14—N5—C15—N6	1.4 (2)
C12—N3—C8—C7	177.04 (16)	C13—N6—C15—N7	178.3 (2)
C10—C7—C8—O5	174.36 (17)	C13—N6—C15—N5	−2.4 (2)
C4—C7—C8—O5	0.2 (3)		

### Hydrogen-bond geometry (Å, º)

**Table:**

*D*—H···*A*	*D*—H	H···*A*	*D*···*A*	*D*—H···*A*
N7—H7*N*1···O5	0.85 (3)	1.96 (3)	2.800 (2)	171 (2)
N7—H7*N*2···O7^i^	0.82 (3)	1.95 (3)	2.749 (2)	165 (3)
N6—H6*N*···O1*W*^ii^	0.84 (3)	2.05 (3)	2.767 (2)	142 (2)
O1*W*—H1*WA*···O6^iii^	0.81 (4)	1.99 (4)	2.792 (2)	166 (4)
O1*W*—H1*WB*···O3	0.79 (4)	2.47 (4)	3.083 (3)	136 (4)
O1*W*—H1*WB*···O8^iv^	0.79 (4)	2.61 (4)	3.080 (3)	120 (4)
C12—H12*C*···O5^iii^	0.96	2.57	3.483 (3)	159
C14—H14*B*···O1^ii^	0.97	2.44	3.270 (3)	144
C16—H16*C*···O5	0.96	2.54	3.248 (2)	131

Symmetry codes: (i) −*x*+3/2, *y*−1/2, −*z*+1/2; (ii) −*x*+1/2, *y*−1/2, −*z*+1/2; (iii) −*x*+1, −*y*+2, −*z*; (iv) −*x*+1/2, *y*+1/2, −*z*+1/2.
